# Custo-Efetividade do Uso do Escore de Cálcio Coronariano na Prevenção Primária enquanto Norteador da Decisão Terapêutica

**DOI:** 10.36660/abc.20230542

**Published:** 2023-12-14

**Authors:** Cristian Rodrigues do Nascimento, Júlio Martinez Santos, Rodrigo Mendes, Johnnatas Mikael Lopes, Pedro Pereira Tenório

**Affiliations:** 1 Universidade Federal do Vale do São Francisco Colegiado de Medicina Paulo Afonso BA Brasil Universidade Federal do Vale do São Francisco – Colegiado de Medicina , Paulo Afonso , BA – Brasil; 2 Irmandade Santa Casa de Misericórdia de São Paulo São Paulo SP Brasil Irmandade Santa Casa de Misericórdia de São Paulo , São Paulo , SP – Brasil

**Keywords:** Fatores de Risco de Doenças Cardíacas, Doença das Coronárias, Tratamento Farmacológico, Calcificação Vascular, Farmacoeconomia

**Ao Editor,**

Lemos com muito interesse o artigo: Custo-Efetividade do Emprego do Escore de Cálcio Coronariano na Orientação para Terapia na Prevenção Primária, na População Brasileira **.** O estudo objetivou avaliar o custo-efetividade do emprego do escore de cálcio na orientação terapêutica para a prevenção primária cardiovascular. As análises foram feitas com base nos dados populacionais do *Multi-Ethnic Study of Atherosclerosis* (MESA), uma coorte composta de 6.814 participantes de diversos centros de estudos dos Estados Unidos. ^1^ As inferências feitas pelos autores são importantes, sobretudo no que tange a identificar fatores relacionados à farmacoeconomia na prevenção de doenças cardiovasculares. Contudo, identificamos que as condutas sugeridas mediante o uso do escore de cálcio (EC) para definir o uso ou não de estatinas pode levar ao viés do tipo atrito, pois há situações clínicas que são necessários mais estudos de imagens sobre o acometimento da estrutura vascular, sobretudo das coronárias para que se consiga definir as condutas posteriores.

É sabido que o comportamento dos segmentos vasculares, sobretudo, arterial, não segue uma regra em diversas doenças, como na hipertensão arterial sistêmica (HAS), diabetes *mellitus* (DM), doença renal crônica (DRC) dialítica e não-dialítica, dislipidemias e obesidade. Assim, percebe-se que a estratificação de risco para indicar a prescrição de estatinas ou a realização do EC pode ter sofrido viés de seleção. Outrossim, de acordo com a literatura, há indícios que indivíduos com moderado ou alto risco cardiovascular que apresentaram EC zero, ainda assim, tinham componentes importantes que levavam a alterações vasculares graves, aumentando o risco cardiovascular. Logo, é de grande valia que se investigue as alterações encontradas não só na presença de calcificações vasculares, pois no estudo de Nurmohamed et al. ^2^ fica claro que há a necessidade de investigar o acometimento vascular antes mesmo da presença de placas calcificadas.

Assim, o EC apenas indica uma estimativa da quantidade de placa aterosclerótica presente, desde que esta contenha pontos de calcificação, não importando o comprometimento da luz vascular. Diante disso, acreditamos que seria imprescindível o uso de uma tecnologia diagnóstica que pudesse ser utilizada para avaliar o comprometimento vascular sem necessitar da presença de calcificações para detectar tais alterações. Desse modo, a angiotomografia de coronárias (ATCC) objetiva não identificar as placas calcificadas, mas consegue mensurar o grau de obstrução do lúmen vascular promovida pela placa aterosclerótica.

Nesse sentido, o estudo de Gabriel et al. ^3^ avaliou a frequência de placa aterosclerótica coronária, assim como seu grau de obstrução e fatores associados em pacientes com EC zero com indicação clínica de ATCC. Em um total de 367 indivíduos, foi encontrada uma frequência de placa aterosclerótica nas coronárias de 9,3%; IC95%, 6,3 – 12,3. Diante disso, levando em consideração o fluxograma proposto pelos autores do artigo em tela e aplicando-o à população deste estudo, ^2^ teríamos 34 indivíduos que não estariam em uso de estatina. Logo, poderíamos agravar o risco cardiovascular desses indivíduos, pois a lesão aterosclerótica poderia continuar em desenvolvimento e inferir mais risco de lesão isquêmica cardíaca.
