# Supplementary material for: Custo-Efetividade do Uso do Escore de Cálcio Coronariano na Prevenção Primária enquanto Norteador da Decisão Terapêutica
Source: Arq Bras Cardiol. 2023 Dec 14;120(11):e20230542. [Article in Portuguese] doi: 10.36660/abc.20230542 (PMC10763693; doi:10.36660/abc.20230542)
Supplement: Translation

# Translation: Cost-Effectiveness of the Use of the Coronary Calcium Score in Primary Prevention as a Guide for the Therapeutic Decision

**Authors:** Cristian Rodrigues do Nascimento, Júlio Martinez Santos, Rodrigo Mendes, Johnnatas Mikael Lopes, Pedro Pereira Tenório

**Affiliations:** 1 Brazil Universidade Federal do Vale do São Francisco – Colegiado de Medicina, Paulo Afonso, BA – Brazil; 2 Brazil Irmandade Santa Casa de Misericórdia de São Paulo, São Paulo, SP – Brazil

**Keywords:** Heart Disease Risk Factors, Coronary Disease, Drug Therapy, Vascular Calcification, Economics Pharmaceutica

**To the Editor,**

We read with great interest the article: “Cost-Effectiveness of Using the Coronary Calcium Score to Guide Therapeutic Decisions in Primary Prevention in the Brazilian Population” The study aimed to evaluate the cost-effectiveness of using the calcium score in therapeutic guidance for primary cardiovascular prevention. The analysis was based on population data from the “ *Multi-Ethnic Study of Atherosclerosis (MESA),”* a cohort of 6,814 participants from different study centers in the United States. ^1^ The inferences made by the authors are important, especially when it comes to identifying factors related to pharmacoeconomics in the prevention of cardiovascular diseases. However, we identified that the procedures suggested using the calcium score (CE) to define the use or not of statins could lead to attrition bias, as there are clinical situations in which more imaging studies on the involvement of the vascular structure are necessary, especially the coronary arteries so that subsequent management can be defined.

It is known that the behavior of vascular segments, especially arterial ones, does not follow a rule in several diseases, such as systemic arterial hypertension (SAH), diabetes mellitus (DM), dialytic and non-dialytic chronic kidney disease (CKD), dyslipidemia and obesity. Thus, it is clear that the risk stratification to indicate the prescription of statins or the performance of CE may have suffered selection bias. Furthermore, according to the literature, there is evidence of cardiovascular risk. Therefore, it is of great value to investigate the changes found in individuals with moderate or high cardiovascular risk who presented zero CS; even so, they had important components that led to severe vascular changes, increasing only in the presence of vascular calcifications, as in the study of Nurmohamed et al. ^2^ it is clear that there is a need to investigate vascular involvement even before the presence of calcified plaques.

Thus, the CE only indicates an estimate of the amount of atherosclerotic plaque present, as long as it contains calcification points, regardless of the compromise of the vascular lumen. Given this, we believe that it would be essential to use a diagnostic technology that could be used to assess vascular impairment without requiring the presence of calcifications to detect such changes. Therefore, coronary tomography angiography (CCTA) aims not to identify calcified plaques but can measure the degree of obstruction of the vascular lumen promoted by the atherosclerotic plaque.

In this sense, the study by Gabriel et al. ^3^ evaluated the frequency of coronary atherosclerotic plaque and its degree of obstruction and associated factors in patients with zero CS with clinical indications for CCTA. In 367 individuals, the frequency of atherosclerotic plaque in the coronary arteries was 9.3%; 95%CI, 6.3 – 12.3. Therefore, considering the flowchart proposed by the article’s authors in question and applying it to the population of this study, ^2^ we would have 34 individuals who would not be using statins. Therefore, we could worsen the cardiovascular risk of these individuals, as the atherosclerotic lesion could continue to develop and infer a greater risk of ischemic cardiac injury.
